# RING-Domain E3 Ligase-Mediated Host–Virus Interactions: Orchestrating Immune Responses by the Host and Antagonizing Immune Defense by Viruses

**DOI:** 10.3389/fimmu.2018.01083

**Published:** 2018-05-22

**Authors:** Yuexiu Zhang, Lian-Feng Li, Muhammad Munir, Hua-Ji Qiu

**Affiliations:** ^1^State Key Laboratory of Veterinary Biotechnology, Harbin Veterinary Research Institute, Chinese Academy of Agricultural Sciences, Harbin, China; ^2^Division of Biomedical and Life Sciences, Faculty of Health and Medicine, Lancaster University, City of Lancaster, United Kingdom

**Keywords:** RING-domain E3 ligases, ubiquitination, innate immunity, adaptive immunity, immune regulators

## Abstract

The RING-domain E3 ligases (RING E3s), a group of E3 ligases containing one or two RING finger domains, are involved in various cellular processes such as cell proliferation, immune regulation, apoptosis, among others. In the host, a substantial number of the RING E3s have been implicated to inhibit viral replication through regulating immune responses, including activation and inhibition of retinoic acid-inducible gene I-like receptors, toll-like receptors, and DNA receptor signaling pathways, modulation of cell-surface expression of major histocompatibility complex, and co-stimulatory molecules. During the course of evolution and adaptation, viruses encode RING E3s to antagonize host immune defense, such as the infected cell protein 0 of herpes simplex virus type 1, the non-structural protein 1 of rotavirus, and the K3 and K5 of Kaposi’s sarcoma-associated herpesvirus. In addition, recent studies suggest that viruses can hijack the host RING E3s to facilitate viral replication. Based on emerging and interesting discoveries, the RING E3s present novel links among the host and viruses. Herein, we focus on the latest research progresses in the RING E3s-mediated host–virus interactions and discuss the outlooks of the RING E3s for future research.

## Introduction

In vertebrates, the immune system is composed of the innate and adaptive components. The innate immune system is a highly effective first-line of defense to detect and fight against invading pathogens, and it is initiated when pattern recognition receptors (PRRs) specifically recognize pathogen-associated molecular patterns (PAMPs) (1, 2). Subsequently, activated PRRs trigger downstream signaling pathways through different adaptor proteins to restrict or eliminate pathogens (3). In case invading pathogens break the innate immunity, the tailor-made adaptive immune responses mount an antigen-specific resistance. Generally, cumulative actions of both innate and adaptive immune systems provide powerful defense against pathogens.

To accomplish such enormous and elaborative defense tasks, the immune system is under strict and precise regulation. One of the most important and extensive mechanisms is the ubiquitination, which plays a crucial role in the immune system (4). Three enzymes are essentially involved in protein ubiquitination including ubiquitin (Ub)-activating enzymes (E1s), Ub-conjugating enzymes (E2s), and Ub-ligating enzymes (E3s) (5). The E3s are the vital components owing to the substrate specificity of the ubiquitination cascade (6). Based on the E3 catalytic core domain, E3s are grouped into three families: the homology to E6AP C-terminus (HECT), the U-box protein families, and the RING-domain E3 ligases (RING E3s) (7). Emerging evidences have shown that the RING E3s play multiple roles in biological processes, including cell proliferation and differentiation (8), immune regulation (9), cancers (10), apoptosis (11), among others. Notably, functions of the RING E3s in regulating immunity system are comparatively broad, and increasing numbers of the novel RING E3s are being reported. In the host, the RING E3s not only positively regulate immune system to amplify the antiviral responses but also negatively regulate immune system to decrease the magnitude and duration of immune responses for appropriate antiviral immunity. These counter-actions of the RING E3s indicate that they orchestrate immune responses at the interface of host–virus interactions.

Since the host and viruses co-evolved over centuries, viruses have acquired tactics to antagonize the functions of the RING E3s at various levels to minimize host antiviral responses. Several studies have shown that viruses encode the RING E3s to facilitate the virus replication. Well-characterized examples include infected cell protein 0 (ICP0), an immediate-early proteins encoded by herpes simplex virus type 1 (HSV-1), which plays important roles during lytic and latent infections (12); NSP1, a non-structural protein encoded by rotavirus (RV), is the chief protein that antagonizes the expression of interferon (IFN) and IFN-stimulated genes (ISGs); K3 and K5, encoded by Kaposi’s sarcoma-associated herpesvirus (KSHV), target plasma membrane proteins for ubiquitination-dependent internalization (13). As mentioned above, proteins act as the RING E3s downregulating host immune responses *via* ubiquitination of several cellular proteins. In addition, recent studies suggest that viruses hijack the host RING E3s to promote viral replication through antagonizing IFN-mediated antiviral responses and ubiquitination of viral proteins.

Until recently, the immunity-related RING E3s have been reported increasingly in response to viral infections. However, potential roles of the RING E3s in host–virus interactions are still in infancy stage. Here, we review the mechanisms of the host RING E3s in regulating immune responses and the functions of the RING E3s encoded or hijacked by viruses in viral replication.

## A Brief History of the RING E3s

**Figure 1:**
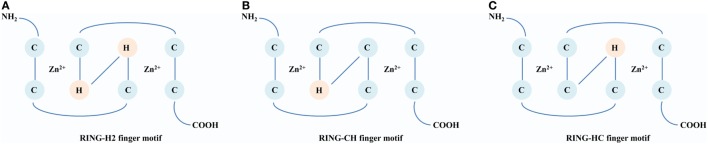
A comparison of structures of the RING domain. Conserved Zn^2+^-binding ligands are shown in circles [cysteine (C) and histidine (H)]. **(A)** RING-H2 finger motif. The histidine residues are in both the fourth and fifth positions within the motif. **(B)** RING-CH finger motif. The cysteine and histidine residues are in the fourth and fifth positions, respectively, within the motif. **(C)** RING-HC finger motif: The histidine and cysteine residues are in the fourth and fifth positions, respectively, within the motif.

The RING E3s, characterized by one or two RING finger motifs, are an extensive family of ligases presenting in various organisms from animals to plants and viruses. There are three milestones that witness the development of the RING E3s. The first identified RING E3 is *Xenopus* oocytes transcription protein TFIIIA, which contains a conserved RING finger motif, Cys–X_2_–Cys–X_9_–_39_–Cys–X_l–3_–His–X_2–3_–Cys–X_2_–Cys–X_4–48_–Cys–X_2_–Cys (X could be any amino acid) (14). In 1994, a 3D structure of the RING domain containing eight conserved cysteine and histidine residues was resolved. It was observed that these residues coordinated two zinc ions to constitute a unique 3D structure known as a “cross-brace” (15). According to the presence of cysteine and histidine residues in the fourth and fifth positions, the RING domain is classified into three subgroups: C_3_HC_4_ (RING-HC), C_3_H_2_C_3_ (RING-H2), and C_4_HC_3_ (RING-CH) finger motifs (Figure 1). These relatively tight and stable structures provide a convenient scaffold on which functional amino acid side chains can be placed (16). In 1999, a major protein (E2) was first found to interact with the RING domain and established foundation that the RING finger proteins are E3 ligases (17). According to different functions of E2s, RING E3s can act as Ubs or Ub-like ligases. Since structure and function of the RING domain have been analyzed, there are burgeoning interests in the RING E3s. Currently, over 600 RING E3s have been reported in humans (18). Many RING E3s are soluble, locating in the cytoplasm and nucleus, and at least 49 RING E3s with hydrophobic regions are predicted to be transmembrane (TM) proteins (19). Furthermore, various domains have been found among the RING E3s, which may enrich their functions and warrant further investigations.

## Classification and Domain Structure of the RING E3s

**Figure 2:**
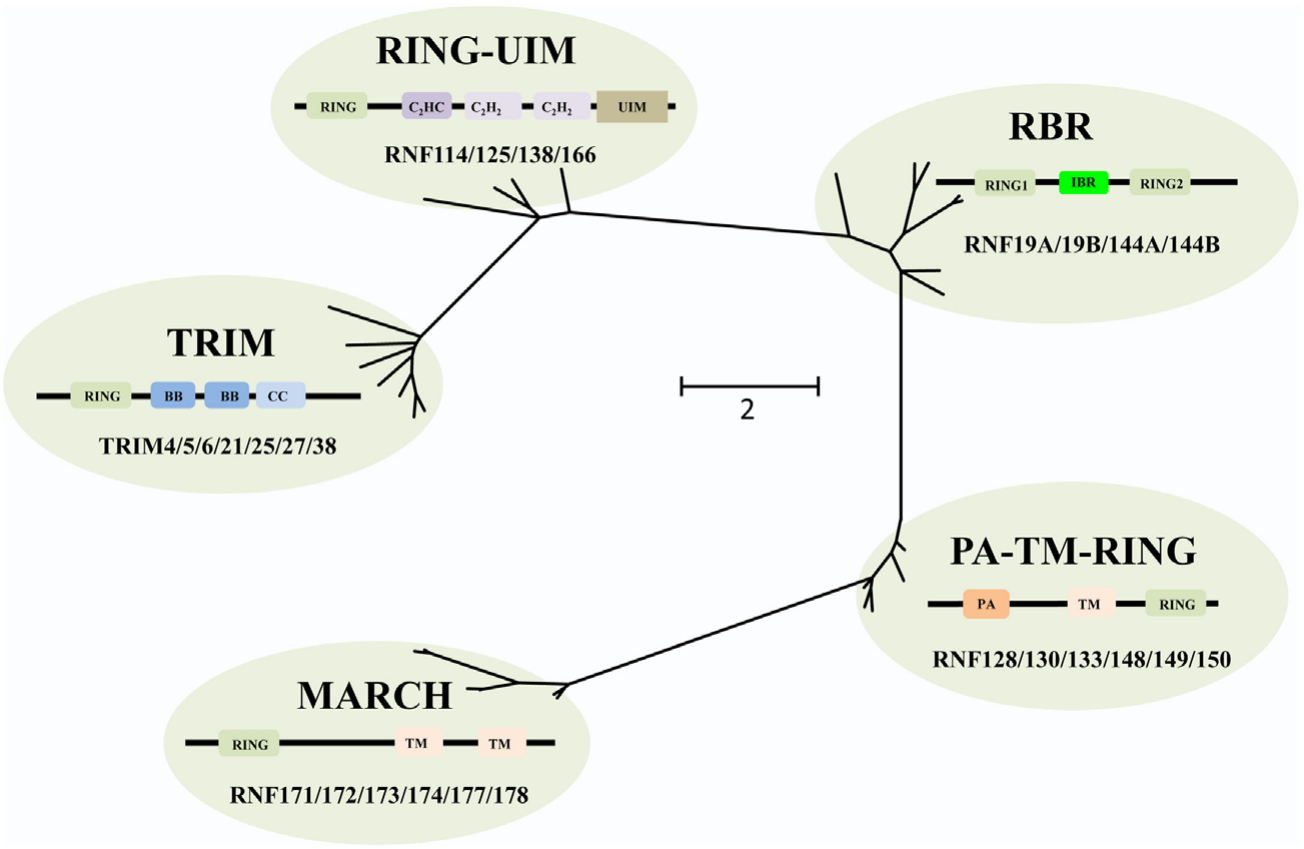
Phylogenetic tree analyses of the RING-domain E3 ligases (RING E3s). Several examples of each type of RING E3s are shown. Each family has its special structure besides the conserved RING domain, which is shown as tawny. For tripartite motif-containing (TRIM) family, two B-box domains (BB), coiled-coiled domain (CC) are colored with dark blue and light blue, respectively. For membrane-associated RING-CH (MARCH) family, transmembrane (TM) domain is pink colored. For PA-TM-RING family, the protease-associated (PA) domain and two TM domains are colored orange and pink, respectively. For RING between RING (RBR) family, in-between-RING (IBR) domain is green colored. For RING-ubiquitin interacting motif (UIM) family, a C_2_HC-type zinc finger, two C_2_H_2_-type zinc fingers, and UIM are colored dark purple, light purple, and brown, respectively. Phylogenetic analysis was performed using MEGA6.

The RING E3s have evolved extensively, and based on phylogenetic analysis the RING E3s can be grouped into five different families, including tripartite motif-containing (TRIM), PA-TM-RING, RING between RING (RBR), membrane-associated RING-CH (MARCH), and RING-Ub interacting motif (UIM) families (Figure 2). All of these five families share a common and conserved RING domain with various unique domains.

The human TRIM family consists of approximately 60 members and shares the RBCC motif, including one RING domain, one or two B-box domains, and one coiled-coiled domain. The C-terminus of TRIM proteins (TRIMs) is intrinsically divergent, allowing them to recruit different sets of corresponding proteins. Therefore, TRIMs are involved in a broad range of biological processes, such as autophagy, immunity, and cancers (20). Recently, increasing evidences have shown that a number of TRIMs act as immunity regulators to inhibit viral replication (21–25).

The MARCH family is structurally characterized by harboring one RING-CH domain and multiple TM domains. This family contains 11 members, i.e., MARCH-1 to -11, of which 9 members are TM proteins, and 2 members, MARCH-7 and -10, have no TM domain (26). Functionally, many MARCH proteins have been identified in viruses, involved in T and B cell functions though downregulating major histocompatibility complex class I (MHC-I) antigen presentation to escape from host defense (27).

The PA-TM-RING family is defined by three conserved domains, the protease-associated (PA) domain, the TM domain, and the RING-H2 domain (28). These proteins belong to the family of endosomal membrane proteins which are characterized by their short life span and low expression in mammals (28). RNF128/GRAIL functions as a gatekeeper of multiple T cell states such as activation, survival, and differentiation (29), while most other members are yet to be characterized.

**Figure 3:**
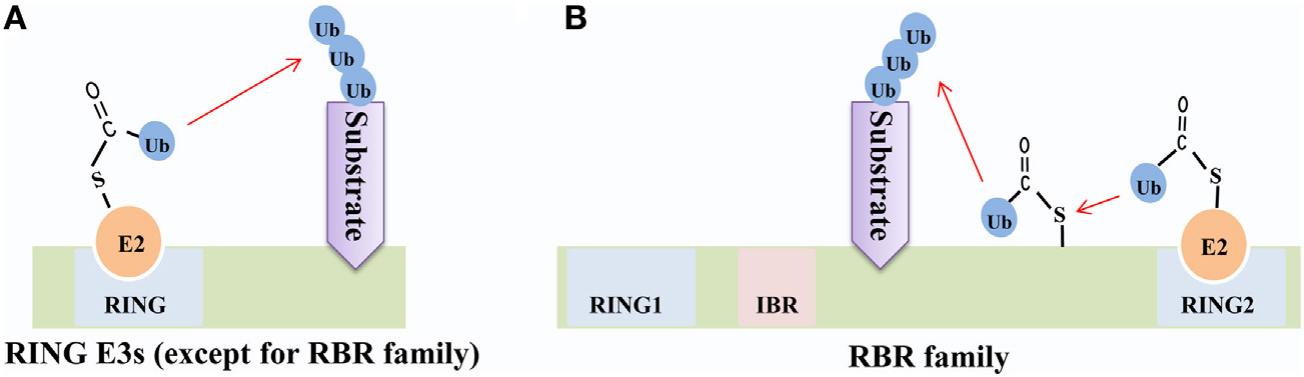
Mechanisms of ubiquitin (Ub) transfer by RING-domain E3 ligases (RING E3s). **(A)** Most RING E3s [except for the RING between RING (RBR) family] employ the classical mechanism that transfers Ub from E2-Ub directly to the substrate. **(B)** The RBR E3s’ N-terminal RING domain (RING1) employs the classical mechanism to facilitate ubiquitination of the substrate, while C-terminal RING domain (RING2) transfers Ub from E2-Ub to generate an E3-Ub intermediate and then transfers Ub to the substrate.

The RBR family (14 members in humans) is characterized by its conservative structure, where two RING domains are connected through an in-between-RING domain (30). These two RING domains perform differential functions; the N-terminal RING domain embracing E3 ligase and acts as a platform to transfer Ub from E2-Ub to substrates, whereas the C-terminal RING domain serves as HECT-like protein that receives Ub from E2-Ub to generate an E3-Ub intermediate. Therefore, it is also called as RING–HECT hybrid (Figure 3) (31, 32). The crystal structure of Parkin, a member of the RBR family, has been resolved and confirms C431 as the Parkin’s cellular active site, supporting the fact that RBR family functions as an RING–HECT hybrid (33, 34).

Finally, the RING-UIM family members share the RING domain, a C_2_HC-type zinc finger, two C_2_H_2_-type zinc fingers, and one UIM-type domain (35). Currently, four members of this family have been reported, named as RNF114, RNF125, RNF138, and RNF166. Albeit the domains of this family have been illuminated, the functional annotation of each domain (except the RING domain) is still unknown. It is reported that the members of this family are associated with IFN-signaling pathways (36–38) and T cell activation (35). Thus, there is a great potential for exploring adaptor protein and regulatory mechanisms for these RING-UIM proteins in the future.

## Host RING E3s as Immune Regulators

### RING E3s in the Innate Immunity

**Figure 4:**
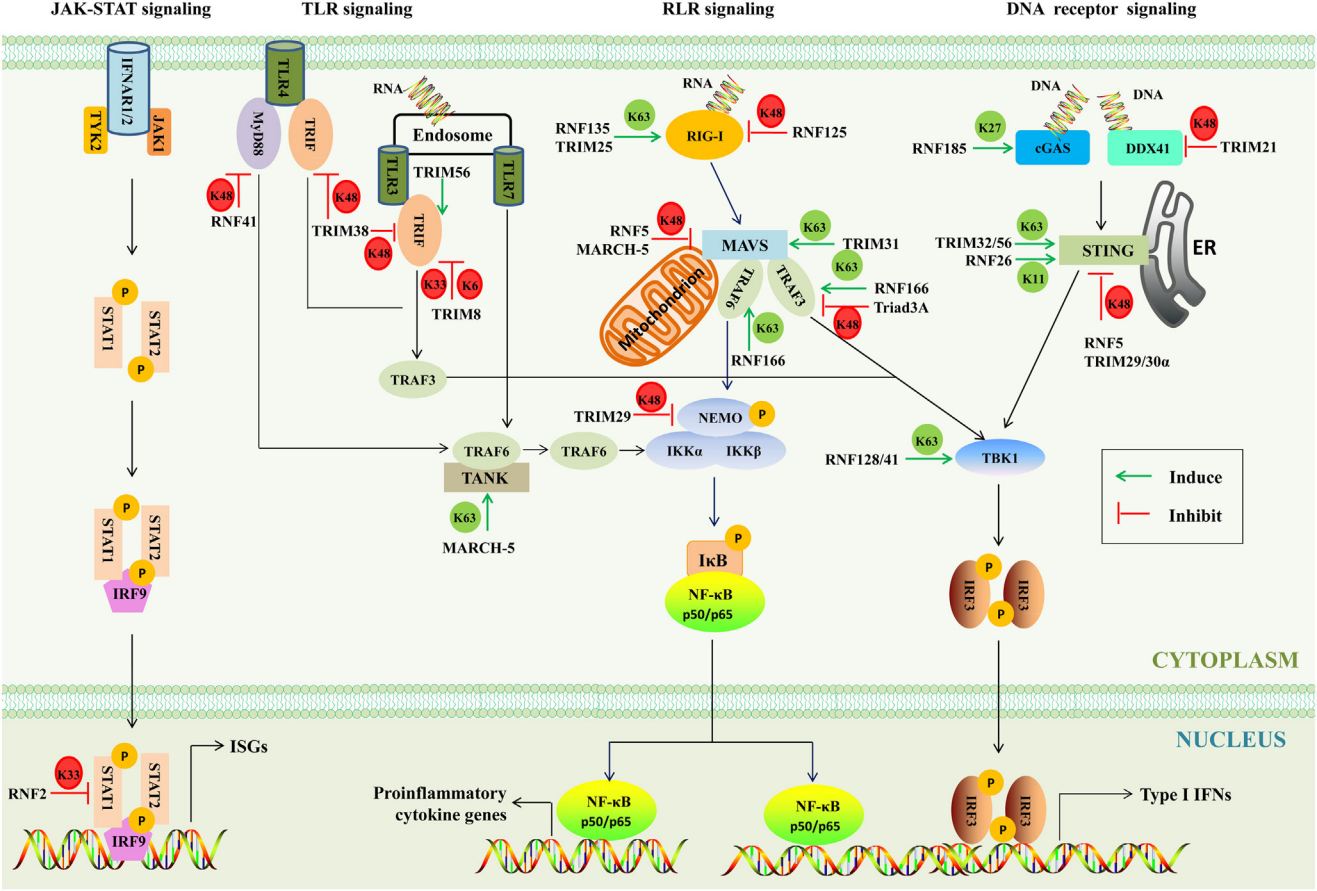
The involvement of RING-domain E3 ligases in mediating innate immunity-associated signaling pathways. The major adaptors are indicated. Abbreviations: RIG-I, retinoic acid-inducible gene I; cGAS, cyclic GMP-AMP synthase; DDX41, DEAD box polypeptide 41; STING, stimulator of interferon genes; TBK1, TANK-binding kinase 1; IRF3 and 9, interferon regulatory factors 3 and 9; MAVS, mitochondrial antiviral signaling; NF-κB, nuclear factor κB; NEMO, NF-κB essential modulator; IκB, inhibitory κB; IKK, inhibitor of κB kinase; TLR3, 4, and 7, toll-like receptors 3, 4, and 7; MyD88, myeloid differentiation primary response protein; TRIF, TIR-domain-containing adaptor protein inducing interferon-β-mediated transcription factor; TRAF, tumor necrosis factor receptor-associated factor; IFN, interferon; TANK, TRAF family member-associated NF-κB activator; IFNAR, type I IFN receptor; JAK-1, Janus kinase 1; TYK2, tyrosine kinase 2; STAT1 and 2, signal transducer and activator of transcription 1 and 2; ISGs, IFN-stimulated genes; TRIM, tripartite motif-containing; MARCH, membrane-associated RING-CH; K6, K6-linked polyubiquitination; K11, K11-linked polyubiquitination; K27, K27-linked polyubiquitination; K33, K33-linked polyubiquitination; K48, K48-linked polyubiquitination; K63, K63-linked polyubiquitination; P, phosphorylation; ER, endoplasmic reticulum.

When pathogens break physical barriers, their PAMPs are detected by PRRs, including retinoic acid-inducible gene I (RIG-I)-like receptors (RLRs), toll-like receptors (TLRs), and DNA receptors (39, 40). As a result, downstream innate immunity signaling pathways are activated and induce various antimicrobial molecules such as IFNs and ISGs to battle the invading pathogens. As a matter of fact, the IFN signaling is tightly regulated by several mechanisms, and one of such mechanisms is ubiquitination (41). A major function of the RING E3s is to regulate the polyubiquitination of target proteins (42). Different lysine residues (K6, K11, K27, K29, K33, K48, and K63)-linked polyubiquitination determines the fate of substrates. It is believed that polyubiquitin chains linked through K48 target substrates for proteasome degradation, while the K63-linked polyubiquitination activates or relocates substrate (43). Up to now, the functions of other types of polyubiquitination remain largely unknown (44). In the host, the RING E3s-mediated multiple ubiquitination orchestrates innate immune responses to ensure optimal virus restriction (Figure 4).

#### RLR Signaling

There are three members of RLRs, including RIG-I, melanoma differentiation associated gene 5 (MDA5), and laboratory of genetics and physiology 2, all of which contain RNA helicase domains sensing viral double-stranded RNA in the cytosol (45). The N-terminal caspase activation and recruitment domain of RIG-I binds to the K63-linked polyubiquitin chains to activate RIG-I (46). RNF135 (47) and TRIM25 (48) were reported to activate RIG-I through transferring the K63-linked polyubiquitination and evoking human innate immunity against RNA virus infection. Ubiquitinated RIG-I and MDA5 induce the prion-like polymerization of mitochondrial antiviral signaling (MAVS), which in turn recruits and activates the E3s such as tumor necrosis factor receptor-associated factor (TRAF) 3 and TRAF6. These E3s then synthesize polyubiquitin chains that are sensed by NF-κB essential modulator (NEMO) (49). TRIM31 binds to inactive MAVS after viral infection and catalyzes the K63-linked polyubiquitination at Lys10, 311, and 461 to further promote aggregation of MAVS and thus enhance IFN-β production and antiviral signaling (50). RNF166, a member of the RING-UIM family, interacts with TRAF3 and TRAF6 *via* its RING domain and enhances the ubiquitination of TRAF3 and TRAF6 to positively regulate RNA virus-triggered IFN-β production (37). A notable feature of the RING E3s is that they tend to form homodimers and heterodimers (51). As mentioned earlier, RNF166 could interact with and activate TRAF3 and TRAF6, which gains insights into the interactions among E3s. Upon linked polyubiquitin chains, NEMO then recruits TANK-binding kinase 1 (TBK1) to form complexes. RNF128 associates with TBK1 and promotes TBK1 kinase activity through conjugation of K63-linked polyubiquitin chains, inducing IFN-regulatory factor (IRF) 3 activation and IFN-β production (52).

By contrast, some RING E3s have been found to negatively regulate innate signaling pathways through mediating the K48- or K33-linked polyubiquitination. RNF125 possesses the ability to conjugate Ub to RIG-I and MDA5 to suppress the RIG-I-mediated signaling pathway (36). Furthermore, MAVS is an important adaptor of the RLR signaling, which is targeted by RNF5 and MARCH-5 for the K48-linked polyubiquitination and degradation (53, 54). The TRAF3 is recruited by MAVS to activate the downstream signaling. Triad3A, a member of RBR family, negatively regulates RLR signaling by targeting TRAF3 for degradation following RNA virus infection (55). NEMO, a key adaptor in both the IRF-mediated type I IFN production pathway and the nuclear factor κB (NF-κB)-mediated proinflammatory signaling pathway, is targeted by TRIM29 at Lys183 by the K48-linked polyubiquitination (56). The type I IFNs bind with their cognate IFN receptors and activate the downstream JAK–STAT signaling pathway, and as a result STAT1/STAT2/IRF9 hybridizes with the target DNA to induce the transcriptional regulation of myriad of ISGs. RNF2 functions *via* K33-linked polyubiquitination at Lys379 of STAT1 in the nucleus to promote STAT1/STAT2 disassociation from DNA, inhibiting type I IFN signaling and antiviral responses (57). Moreover, it was also reported that RNF34, RNF37, RNF41, RNF111, RNF144b, RNF145, RNF157, and RNF158 could inhibit the antiviral responses. However, the functions of these RING E3s in the regulation of IFN signaling remain largely unknown (57).

Intriguingly, RNF114 has been reported to positively and negatively regulate IFN-associated signaling pathways. It has been revealed that overexpression of RNF114 enhances NF-κB and IRF3 reporter activity and positively regulate mRNA levels of types I and III IFNs. It is therefore speculated that RNF114 may modulate the RIG-I or MDA5 signaling pathway (58). On the contrary, recent studies have shown that RNF114 acts as negative regulator of RLR signaling (38, 59). It has been argued that RNF114 could negatively regulate the RLR signaling through degrading MAVS or stabilizing A20 and IκBα. Furthermore, RNF114 was identified as a psoriasis-susceptibility gene (60). Therefore, further investigations are warranted to assess the dynamic and plasticity in regulatory roles of RNF114.

#### TLR Signaling

Toll-like receptors are single transmembrane proteins, containing leucine-rich repeats to recognize PAMPs and cytosolic toll/IL-1 receptor (TIR) domain to transduce signals to downstream adaptors, including myeloid differentiation primary response protein (MyD88) and TIR-domain-containing adaptor protein inducing interferon-β-mediated transcription factor (TRIF) (61). The MyD88-dependent pathway uses the TAK1 kinases to activate transcription factor NF-κB and AP-1, while the TRIF-dependent pathway through a cascade of TRIF, the TBK1 kinase, the adaptor TRAF3, and the transcription factor IRF3 results in the production of type I IFN (62, 63). RNF41 plays dual roles; on one hand, it can mediate K48-linked polyubiquitination and degrade MyD88 to inhibit MyD88-dependent pathway, and on the other hand, RNF41 promotes activation of TBK1 and IRF3 through mediating the K63-linked polyubiquitination of TBK1 to potentiate TRIF-dependent pathway (64). Furthermore, TRIM56 interacts with TRIF contributing to TLR3-mediated IRF3 activation, and the effector is independent of the E3s (65). TRIM38 mediates K48-linked polyubiquitination at Lys228 and degrades TRIF to negatively regulate TLR3/4-mediated signaling pathway (66). TRIM8 inhibits the TLR3/4-mediated signaling pathway by catalyzing K6- and K33-linked polyubiquitination of TRIF and disrupting the TRIF-TBK1 interaction (67). TRAF family member-associated NF-κB activator (TANK) is a negative regulator of the TLR7 signaling pathway by suppressing TRAF6 ubiquitination (68). MARCH-5 catalyzes the K63-linked polyubiquitination of TANK at Lys229, 233, 280, 302, and 306. This modification releases the inhibitory effects of TANK on TRAF6 (69).

#### DNA Receptor Signaling

Many sensors of cytosolic viral DNA have been identified, including cyclic GMP-AMP synthase (cGAS) (70), the helicase DEAD box polypeptide 41 (DDX41) (71), absent in melanoma 2 (72), DNA-dependent activator of IRFs (73), and interferon gamma-inducible protein 16 (74). Both DDX41 and cGAS mediate stimulator of interferon genes (STING)-dependent type I IFN responses (71, 75). RNF185 specifically catalyzes the K27-linked polyubiquitination of cGAS, promoting its enzymatic activity (76). In myeloid dendritic cells and monocytes, TRIM21 negatively regulates the type I IFN response by promoting the ubiquitination and degradation of DDX41 (77). Once activated by cytosolic DNA signaling, STING, as a crucial signaling adaptor, recruits and activates the TBK1 kinase, which then activates the IRF3 transcription factor to induce type I IFNs (78, 79). It has been reported that multiple mechanisms are involved in the STING-mediated immune response. One such mechanism is that TRIM56 and TRIM32 catalyze the K63-linked polyubiquitination of STING, leading to its activation (80, 81). Another is that RNF5, TRIM29, and TRIM30α promote the K48-linked polyubiquitination of STING to dampen the cytosolic virus-triggered immune responses (82–84). In addition, RNF26 catalyzes K11-linked polyubiquitination of STING, which unlocks its K48-linked polyubiquitination and prevents its proteasome degradation (85).

### RING E3s in the Adaptive Immunity

When pathogens conquer the innate immunity, adaptive immunity works under the control of humoral and cellular immunity. In the cellular immunity, T cell activation requires two elements: T cell’s receptors (TCRs) specially recognize MHC-bounded pathogen peptides and T cell ligands bind with co-stimulation factor provided by activated antigen presenting cells (86). Interestingly, the RING-UIM, MARCH, and TRIM families stand out in regulating adaptive immunity. The RING-UIM and TRIM families positively regulate T cell activation, whereas the MARCH family negatively regulates T cell activation through degrading the cell-surface expression of immune regulators. Consequently, the RING E3s work synergistically to fine tune the adaptive immunity.

To defend against invading pathogens, the RING E3s enhance the expression of co-stimulatory factors to further amplify the function of T cells and to inhibit the viral replication. As discussed earlier, the RING-UIM family not only plays a role in innate immunity but also in adaptive immunity. RNF125 has been reported to positively regulate T cell activation, since silencing of the RNF125 expression inhibits T cell activation in response to TCR cross-linking (87, 88). Recently, other members including RNF166 and RNF114 were reported subsequently. The overexpression of RNF166 in primary T cells and Jurkat T cells induces over 2-fold increase of CD69, a T-cell activation marker, suggesting that RNF166 is also a positive regulator of T cell activation (89). RNF114 is a novel positive regulator of T cell activation, in which the C_2_H_2_ domain is involved in this process (90). At present, the functions of RNF138 are elusive; however, based on the domain similarity among the RING-UIM family, RNF138 probably performs a similar role in T cells.

In contrast with the RING-UIM family, the MARCH proteins function as negative regulator in adaptive immunity. The detailed roles of MARCH proteins in adaptive immunity have been reviewed recently (26, 27). More than 600 RING E3s have been identified in humans. Since MARCH proteins prefer to target MHC complexes, possible explanations are summarized as follows: first, locational proximity, MHC-II could be downregulated by MARCH-1 and -8 (91, 92), it could be explained that the MHC-II was found proximal to the RING domain of MARCH-1 and -8 on chromosome 6 (93). Second, structural similarity, MARCH proteins show overlapping motif with most of MHC complexes and some co-stimulatory molecules. MHC-II heterodimers is mediated by G–X_3_–G (X could be any amino acid) motifs in their TM helices (94), and the G–X_3_–G motifs are also present in TM helices of the MARCH proteins. G–X_3_–G motifs are involved in the selection and recognition of other membrane proteins as substrates for ubiquitination, thus MHC complexes may be recognized by MARCH proteins (95).

Recently, TRIMs were identified to play a critical role in adaptive immunity, this topic is referred to the recent excellent review (25). TRIM21 is a well-characterized TRIM protein, which acts as both immune sensor and effector to promote an integrated antiviral response (96). TRIM21 shows remarkably broad antibody specificity since the C-terminal PRYSPRY domain binds with high affinity to the Fc portion of IgG, IgM, or IgA (97). When viruses coated with these antibody isotypes enter the cytosol, TRIM21 rapidly recruits and efficiently neutralizes viruses for proteasome degradation (98).

So far, most studies on the RING E3s have focused on their roles as antiviral factors to indirectly restrict virus replication by orchestrating immune responses. In addition, they can also directly restrict virus replication by interfering with the key steps of the virus life cycle. RNF125 could downregulate HIV-1 transcription through proteasome during the viral transcription (99). The MARCH-8, highly expressed in terminally differentiated myeloid cells, targets HIV-1 envelope glycoproteins and reduces their incorporation into virions (100). TRIM22 catalyzes influenza A viruses (IAV) nucleoprotein ubiquitination and degradation in a proteasome-dependent manner to inhibit IAV replication (101). TRIM52 is a novel antiviral TRIM protein, and it exerts antiviral activity against Japanese encephalitis virus infection by targeting and degrading viral NS2A (102). The studies about direct interactions between host RING E3s and viruses are limited. Therefore, further studies are required to deepen the understanding of host RING E3s’ impacts on viruses.

## Viruses Employ RING E3s to Interfere with the Host Immune Response

**Figure 5:**
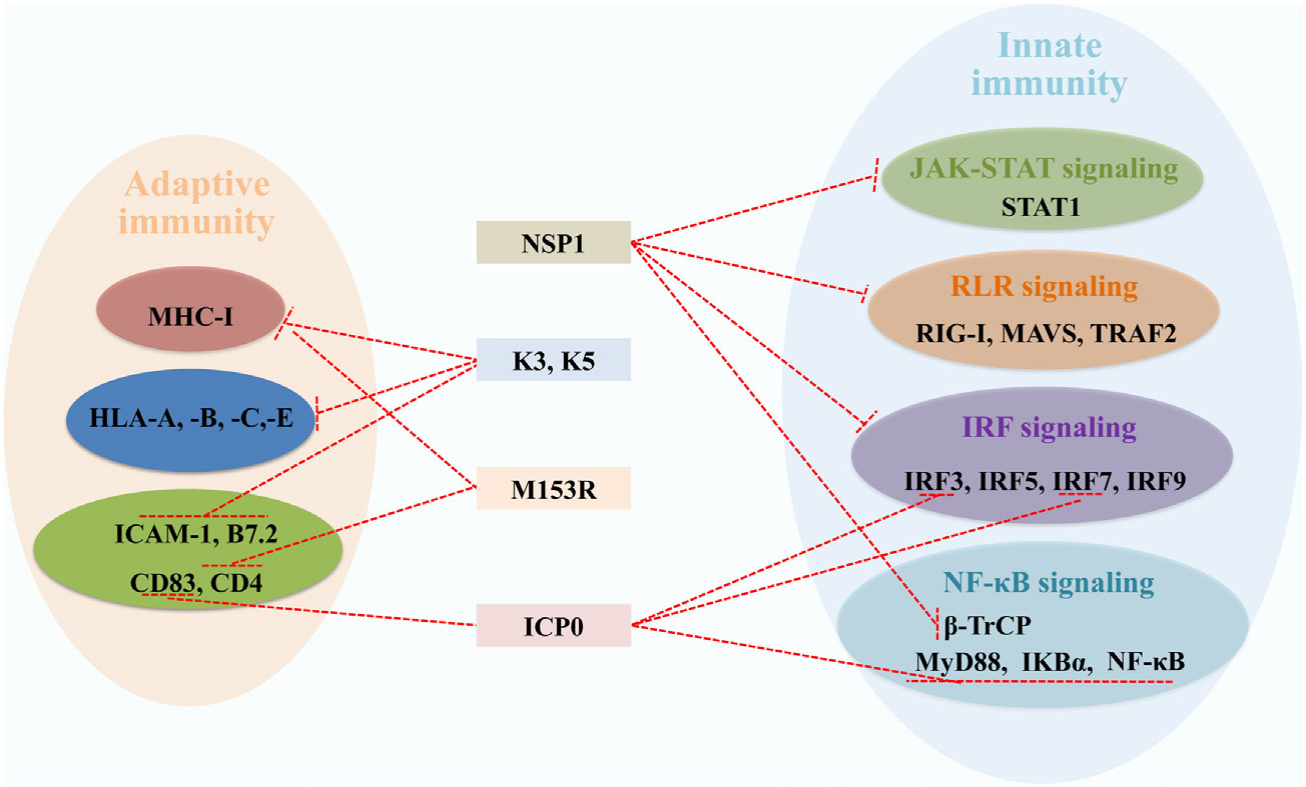
Viral RING-domain E3 ligases counteract components of the host innate and adaptive immunity by targeting cellular proteins, which are shown with a T-shaped line in red. Abbreviations: NSP1, the non-structural protein 1 of rotavirus; K3 and K5, the K3 and K5 proteins of Kaposi’s sarcoma-associated herpesvirus; M153R, the M153R protein of myxomavirus; ICP0, the infected cell protein 0 of herpes simplex virus type 1. STAT1, signal transducer and activator of transcription 1; RIG-I, retinoic acid-inducible gene I; MAVS, mitochondrial antiviral signaling; TRAF2, tumor necrosis factor receptor-associated factor 2; IRF3, 5, 7, and 9, interferon regulatory factors 3, 5, 7, and 9; β-TrCP, β-transducin repeat-containing protein; MyD88, myeloid differentiation primary response protein; NF-κB, nuclear factor κB; IKBα, inhibitory κB alpha; MHC-I, major histocompatibility complex class I; HLA-A, -B, -C, and -E, HLA class I histocompatibility antigens A, B, C, and E; ICAM-1, intercellular adhesion molecule 1; B7.2, cytotoxic T-lymphocyte-associated antigen 4 counter-receptor; CD83 and 4, CD_antigens 83 and 4.

In the host, the RING E3s-mediated posttranslational attachment of Ub to proteins inhibits virus replication. Conversely, some viruses have adapted to counteract the RING E3s-mediated immune defense. As it is well known that the RING E3s play a direct catalytic role in protein ubiquitination, increasing evidence indicates that viruses can employ RING E3s’ catalytic activity to redirect the ubiquitination machinery of the host and viruses. Here, we discuss two strategies that viruses have developed to counteract antiviral defense. One is that viruses encode RING E3s to target and degrade host immune proteins by proteasome or lysosome, including ICP0, NSP1, K3, and K5 (Figure 5). Another is that viruses hijack host’s RING E3s to interfere with IFN-mediated antiviral responses and to positively influence specific steps of the replication cycle *via* ubiquitination of viral proteins. The fact that the RING E3s are targeted by viruses for immune evasion further highlights their important roles in protecting the host against viral infections.

### Viruses Encode RING E3s to Antagonize Antiviral Responses

Herpes simplex virus type 1 E3 ligase ICP0, with an RING domain at the N-terminus, can direct several cellular targets for proteasome degradation, allowing the virus to counteract cellular immune responses (103). Considering ICP0’s multifunction to counteract immune responses, HSV-1 is regarded as an IFN-resistant virus. The specific roles of ICP0 in innate and adaptive immune responses have been discussed elsewhere (104). In innate immunity, ICP0 could inhibit IRF3, IRF7, MyD88, IκBα, and NF-κB proteins depending on the RING domain (105–107). In adaptive immunity, DCs upregulate the surface molecule CD83 during its maturation, and ICP0 induces CD83 degradation independent of its E3 ligase and Ub machinery (108, 109).

The RV non-structural protein NSP1, with a conserved N-terminal RING-like domain and a variable C-terminal substrate-targeting domain, is an IFN antagonist. NSP1 can block the transcription of IFN-α/β by targeting RIG-I, tumor necrosis factor receptor-associated factor 2, MAVS, IRF3, IRF5, IRF7, and IRF9 for proteasome degradation (110–113). Recently, NSP1 was reported to employ a novel mechanism to antagonize IFN signaling by degrading β-transducin repeat-containing protein, which is necessary for activation of NF-κB (114–116). The phosphorylation of STAT1 at Y701 is necessary for the formation of a heterotrimeric complex of STAT1, STAT2, and IRF9 (117). NSP1 acts as an antagonist of IFN-mediated STAT1 Y701 phosphorylation to inhibit STAT1 activation, which does not require the RING domain (118). Further studies are needed to learn how NSP1 targets and induces degradation of different and critical proteins of the IFN induction cascade (119).

The KSHV proteins K3 and K5, with an RING-CH domain at the N-terminus, are highly homology with the MARCH family in structure and functions (120). As mentioned before, MARCH proteins regulate cell-surface receptors *via* ubiquitination to block antigen presentation in adaptive immunity, such as MHC-I and some costimulatory molecules. Similarly, K3 and K5 also downregulate the surface expression of MHC-I molecules *via* endocytosis and degradation (121). Moreover, K5 targets costimulatory molecules intercellular adhesion molecule 1 (122) and B7.2 by lysosomal degradation (123). There are two major differences between K3 and K5. First, K3 and K5 show different HLA allotype specificities, K3 downregulates HLA class I histocompatibility antigens A, B, C, and E (HLA-A, -B, -C, and -E), while K5 effectively targets HLA-A and -B, weakly HLA-C and is unable to target HLA-E (124). Second, K3 primarily suppresses cell-mediated immunity, whereas K5 mainly suppresses cytokine-mediated immunity (125). Myxomavirus M153R is the homologous gene product of K3 and K5, characterized by an amino-terminal PHD/LAP domain, targets MHC-I and CD4 by lysosomal pathway, thus effectively escaping from immune recognition (126).

### Viruses Hijack Host RING E3s to Antagonize Antiviral Responses

Besides encoding RING E3s, viruses also hijack the host’s RING E3s to enhance their own replication. The host’s RING E3s that control the immune response pathways are often targeted by viral gene products to counteract their functions. The Ebola virus (EBOV) VP35, a cofactor of the viral polymerase, plays a critical role in viral replication (127). TRIM6 interacts with VP35 and promotes VP35 ubiquitination at Lys309, enhancing the VP35-mediated polymerase activity and virus replication. Therefore, TRIM6 is a host factor hijacked by EBOV-VP35 for promoting viral replication (128). As described in Section “RLR Signaling,” RNF125 mediates the degradation of RIG-I, which in turn downregulates the expression of IFNs. The human bocavirus VP2 interacts directly with RNF125 and inhibits the RNF125-mediated RIG-I degradation, which breaks the balance of IFNs controlled by both positive and negative regulators (129). Yellow fever virus (YFV) inhibiting the type I IFN signaling through a unique mechanism that involves the NS5–STAT2 interaction. This interaction requires IFN-induced tyrosine phosphorylation of STAT1 and the K63-linked polyubiquitination at a lysine in the N-terminal region of YFV NS5. It is reported that TRIM23 promotes YFV NS5 ubiquitination (130). TRIM25 and RNF135 mediate the K63-linked ubiquitination of RIG-I to facilitate type I IFN production and antiviral immunity. The IAV NS1 protein inversely targets TRIM25 and RNF135 for the inhibition of RIG-I ubiquitination and antiviral IFN production (131, 132). It remains to be seen whether other host RING E3s that are targeted by viruses may also directly enhancing viral replication *via* ubiquitination of viral proteins.

## Concluding Remarks and Future Prospects

The RING E3s-mediated orchestration of the immune system has contributed significantly to our understanding of host immune regulation. The RING E3s are divided into five subfamilies, each of which has its own special functions. The RING-UIM family is related to T cell activation, the MARCH family has been identified to inhibit antigen presentation through degrading cellular receptors and costimulatory molecules, and the TRIM family restricts virus replication through direct interactions with viral proteins. At present, there are few reports on RBR and PA-TM-RING family. Therefore, the identification of additional interacting proteins or substrates for RBR and PA-TM-RING families will uncover the novel functions of the RING E3s.

Although the functions and mechanisms of RING E3s-mediated immunity are continuously being reported, there are several fundamental questions that require future research. First, one of the most complex and challenging aspect is to elucidate how the RING E3s are activated upon virus infection and how their expression and localization are regulated during the stimuli in different cell types. Second, it is known that the RING domain credits RING E3s’ powerful E3 ligase function, while the function of other domains is poorly understood, such as C_2_H_2_ and C_2_HC domains of the RING-UIM family. Third, as discussed in this review, it is obvious that multiple substrates can be targeted by the same RING E3, while multiple RING E3s can target the same substrate. The significance of RING E3s “redundancy” in substrate ubiquitination is poorly understood, which leaves a possibility that RING E3s may act as a cross talk to form an immunity-regulating network. In addition, the RING E3s exert various functions in a broad range of pathways such as carcinogenesis and autophagy. It would be important to consider the RING E3s’ functions as hubs connecting different signaling pathways or different systems. A deep understanding of Ub signaling would be extremely valuable for the future development of treatments for immune and inflammatory diseases.

Currently, most studies have primarily been focused on host RING E3s roles as antiviral factors, while RING E3s encoded or hijacked by viruses for evading the immune responses are largely undiscovered, the identification and characterization of these RING E3s has significantly advanced our understanding of persistent infection. It is generally known that ubiquitination can be reversed by deubiquitination enzymes (DUBs). DUBs can rescue proteins from Ub-mediated degradation and reverse the related biological process by removing Ub chains from the substrate. Novel DUBs and inhibitors of these viruses-encoded RING E3s identified will prevent viruses from escaping host defense.

Taken together, the RING E3s-regulated immunity provides a novel perspective to better understand the host–virus interplay. Further detailed investigations are needed using the RING E3s as targets for the prevention and control of recurrent viral diseases.

## Author Contributions

YZ and L-FL are the major contributors of the review. H-JQ and MM conceived and revised the paper.

## Conflict of Interest Statement

The authors declare that the research was conducted in the absence of any commercial or financial relationships that could be construed as a potential conflict of interest.
